# An automated identification and analysis of ontological terms in gastrointestinal diseases and nutrition-related literature provides useful insights

**DOI:** 10.7717/peerj.5047

**Published:** 2018-07-26

**Authors:** Orges Koci, Michael Logan, Vaios Svolos, Richard K. Russell, Konstantinos Gerasimidis, Umer Zeeshan Ijaz

**Affiliations:** 1 Human Nutrition, School of Medicine, College of Medical, Veterinary and Life Sciences, University of Glasgow, Glasgow, UK; 2 Infrastructure and Environment Research Division, School of Engineering, University of Glasgow, Glasgow, UK; 3 Department of Paediatric Gastroenterology, Hepatology and Nutrition, Royal Hospital for Children, Glasgow, UK

**Keywords:** Ontology, Inflammatory bowel disease, Text mining, Ecological statistics, Human nutrition, Ordination, Gastrointestinal disease, Crohn’s disease, Coeliac disease, Ulcerative colitis

## Abstract

With an unprecedented growth in the biomedical literature, keeping up to date with the new developments presents an immense challenge. Publications are often studied in isolation of the established literature, with interpretation being subjective and often introducing human bias. With ontology-driven annotation of biomedical data gaining popularity in recent years and online databases offering metatags with rich textual information, it is now possible to automatically text-mine ontological terms and complement the laborious task of manual management, interpretation, and analysis of the accumulated literature with downstream statistical analysis. In this paper, we have formulated an automated workflow through which we have identified ontological information, including nutrition-related terms in PubMed abstracts (from 1991 to 2016) for two main types of Inflammatory Bowel Diseases: *Crohn’s Disease* and *Ulcerative Colitis*; and two other gastrointestinal (GI) diseases, namely, *Coeliac Disease* and *Irritable Bowel Syndrome*. Our analysis reveals unique clustering patterns as well as spatial and temporal trends inherent to the considered GI diseases in terms of literature that has been accumulated so far. Although automated interpretation cannot replace human judgement, the developed workflow shows promising results and can be a useful tool in systematic literature reviews. The workflow is available at https://github.com/KociOrges/pytag.

## Introduction

The volume of biomedical literature in electronic format has grown exponentially over the past few years (Hunter & Cohen, 2006). With the latest count of 27 million in 2017, PubMed search engine can navigate the MEDLINE database of references and abstracts on life and biomedical sciences using key concepts. Lately, ontology-driven annotation of data has become increasingly important, especially in the biomedical domain (Bodenreider & Stevens, 2006; Lambrix et al., 2007). Ontologies describe controlled dictionaries of words on a given theme and contain axioms that define terms in description logic. By text-mining published abstracts and grouping words used into existing ontologies, it is possible to complement the demanding task of manual management, interpretation and analysis of the vast amount of available research. Recently, Cole et al. (2016) proposed goldi, an open source tool for performing a semi-automated identification of gene ontology in free form biomedical literature provided by the user. Furthermore, RISmed (https://github.com/skoval/RISmed), an R package, is also suggested for extracting bibliographic content from NCBI databases including PubMed although it does not provide any ontology-based text-mining. In both cases, there is a lack of emphasis on the downstream statistical analyses. Previously, we formulated a pipeline called seqenv (Sinclair et al., 2016) and its taxa-centric extension (Ijaz et al., 2017) through which short DNA sequences can be aligned against the NCBI reference databases to extract *Environmental Ontology* (Buttigieg et al., 2013) from the metadata (*Isolation Source* field or relevant PubMed abstracts) associated with the matches. Although prima facie, one might argue that an abstract is not a full paper, it is still a useful piece of information and with a great number of such short texts (typically in thousands), seqenv and its extension did indeed show potential in environmental source tracking of DNA sequences. Using the same principle that we applied to sequencing data, in this paper, we developed a new workflow that automatically annotates PubMed abstracts with rich ontological terms. This can be applied to any disease conditions, as well as allowing the user to perform the same search longitudinally, to highlight changes in a particular area. Downstream data analysis employing ecological statistics is then performed to allow the investigator to interrogate patterns in the context of ontological terms and identify differences between chosen disease groups as well as secular developments within each of these.

This methodology is useful because one not only gets a historical perspective by exploring trends of how the research in a specific topic evolves over a period of time but can also use this information to predict where a particular literature theme is heading. Such an approach can be helpful for systematic reviews as it benefits from the ability to inspect very large number of studies and rapidly annotate thousands of articles with rich metadata in a time-efficient manner of few minutes. It can also reduce the amount of information to manageable sets from which it is easier to infer patterns and trends. In addition, statistical analysis of metadata from multiple ontologies can capture additional details of the content of a research paper and reveal patterns about topics differentiating between test and control groups, something not possible with a traditional, manual approach applied in systematic reviews.

In this paper, we propose a workflow to annotate journal abstracts from nutrition-related literature relevant to two main types of Inflammatory Bowel Diseases (IBDs), namely, *Crohn’s Disease* (CD) and *Ulcerative Colitis* (UC); and two other Gastrointestinal (GI) conditions, *Coeliac Disease* (CCD) and *Irritable Bowel Syndrome* (IBS) where it was assumed a priori these will stand out in terms of nutrition-related terms from the former. We were particularly interested in the subset of papers that covered aspects related to nutrition, using one or more of the following search keywords: *Diet*, *Food,* and *Nutrition*. We hypothesized that: (a) distinct clustering will be observed in nutrition-related terms between the IBD and non-IBD groups; (b) there will be a minimal overlap and close proximity of closely associated conditions on an ordination diagram (beta diversity measure) to suggest that specific ontological terms (e.g., underlying aetiology and dietary factors) are differentiating or converging to similar set of principles; (c) we will be able to pick up nutrition terms that have gained/lost interest in the disease groups (“V” or “inverted-V” shape curves over time); and (d) pinpoint exact location in time when underlying research in terms of nutrition has shifted from exploration (high variability in terms) to exploitation (convergence to certain terms).

## Materials and Methods

### Search strategy for GI diseases and nutrition-related literature

**Figure 1:**
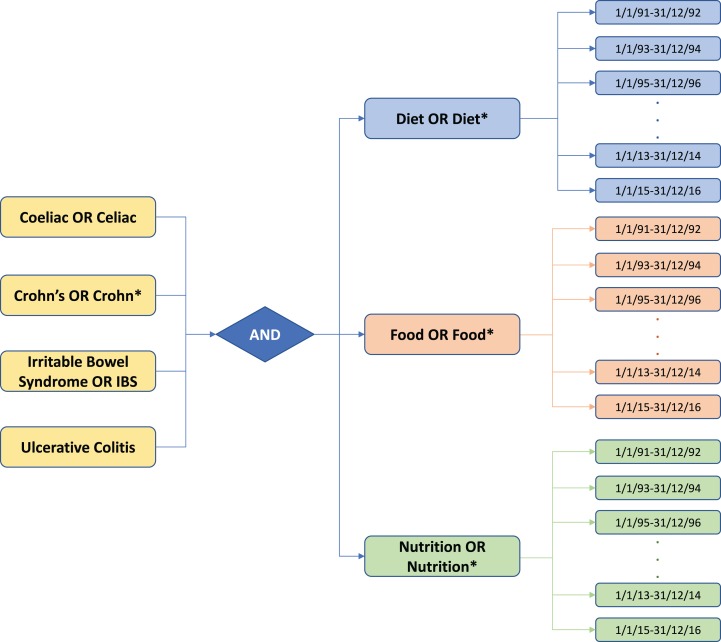
Schematic of the keywords searched in PubMed search engine for the gastrointestinal diseases and nutrition-related literature. Twelve possibilities (4 × disease groups by 3 × nutritional categories) were searched in a 26-year timeline. The returned abstracts were grouped together in pairs of years and collated for a given group. Note that asterisk * are part of the keyword searches in PubMed.

The abstracts used for analysis were retrieved from PubMed database using the list of keywords described in Fig. 1, using a time frame from 1991 to 2016 (searches were performed in July 2017). Composite keywords were constructed through Boolean logic, with four by three possibilities (four disease groups × three nutritional keywords, yielding twelve possible combinations). The returned abstracts were then grouped together in pairs of years, extracted and stored in external files, using the “Citation Manager” function in MEDLINE (tagged) format. The complete search for all the possible combinations from 1991 to 2016, yielded a total number of 24,559 PubMed abstracts. These were then imported into EndNote^®^ X7 citation management software to export them in BibTeX format (input format for our software), where every abstract was described by a number of records including the PubMed ID, i.e., a unique identifier used in PubMed and assigned to each article record when it enters the PubMed system. In total, 156 BibTeX files were generated for all the possible combinations of composite keywords and pairs of years (i.e., 12 possibilities in a 26-year timeline).

### Annotation process

**Figure 2:**
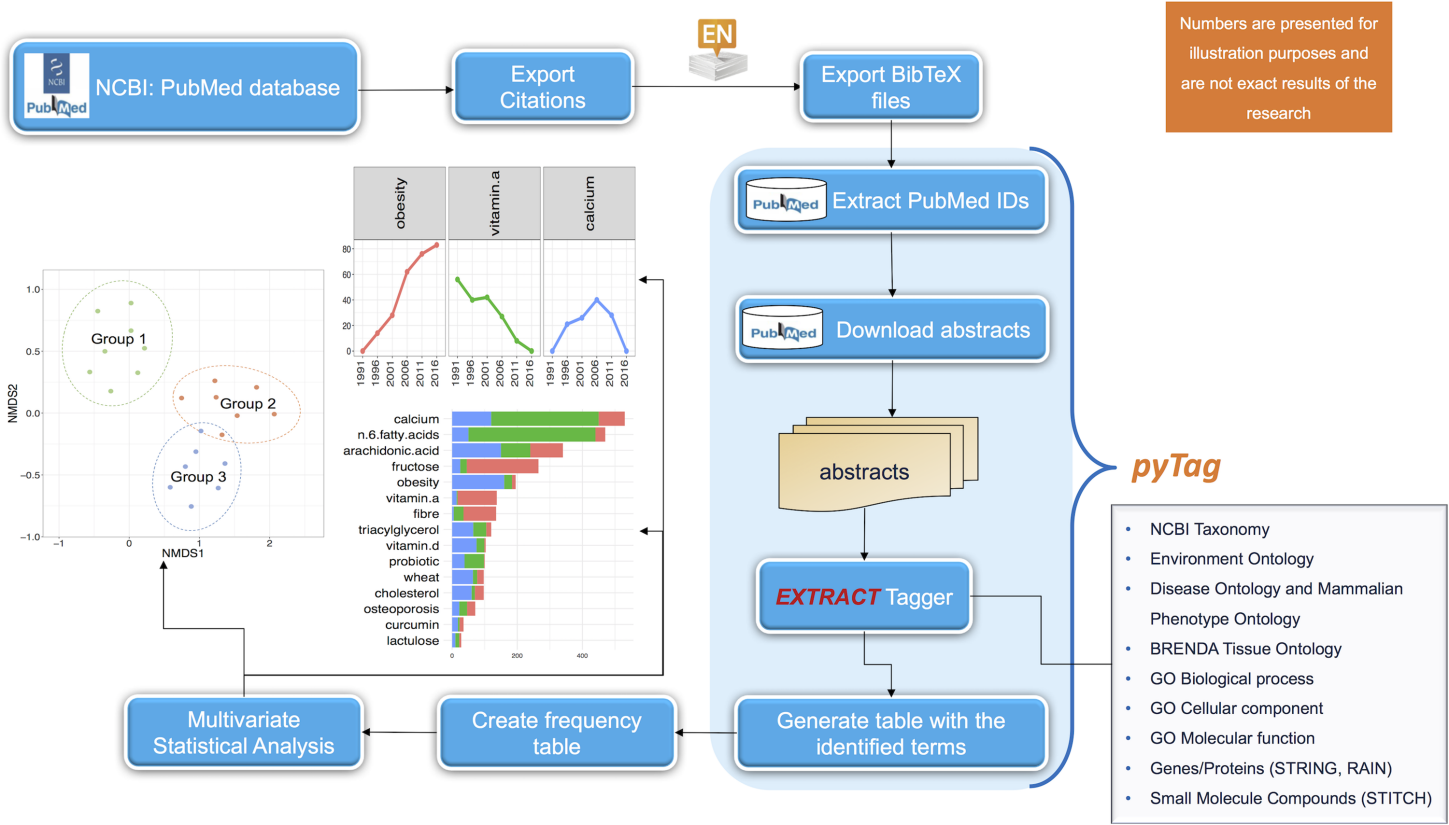
Schematic of the workflow for the automated identification and analyses of ontological terms in literature data. The abstracts returned from a keyword search in PubMed database are extracted and then processed with the pyTag workflow, where all the ontological terms are listed and annotated. After the annotation, a frequency table of the identified terms is generated and next subjected to statistical analysis.

The BibTeX files were then processed by a custom written script called pyTag that accepts these files in a given folder as input (using --input_dir) and supports nine ontologies inherited from EXTRACT 2.0, a custom named entity recognition system (Pafilis et al., 2017) as a switch to the program, with --onto_types all specifying all of them. In general, one is at liberty to choose a subset of these ontologies in a given piece of text. These supported ontologies can recover mentions for: organisms (*NCBI Taxonomy* using --onto_types -2; Federhen, 2011); environments (*Environment Ontology* using --onto_types -27; Buttigieg et al., 2016); diseases and phenotypes (*Disease Ontology* also incorporating *Mammalian Phenotype Ontology* using --onto_types -26; Kibbe et al., 2014; Smith & Eppig, 2012); tissues and cell lines (*BRENDA Tissue Ontology* using --onto_types -25, Placzek et al., 2016); biological processes (--onto_types -21), cellular components (--onto_types -23) and molecular functions (--onto_types -22) using the *Gene Ontology* (The Gene Ontology Consortium, 2015); genes and proteins (STRING and RAIN databases using --onto_types 0; Szklarczyk et al., 2016; Junge et al., 2017); and small molecule compounds (STITCH database using --onto_types -1, Szklarczyk et al., 2015). pyTag then uses these ontologies on the abstracts recovered from the NCBI database using the associated PubMed IDs. After the annotation of all the relevant abstracts, the resulting frequency of the identified *terms* (a formal textual representation for a given concept including all its possible synonyms identifiable by a unique ID for a given ontology) are converted to a two-dimensional abundance table for multiple search criteria, with enough replicates per search to ensure that ecological statistics including alpha and beta diversities can be calculated as well as differential analysis can be performed. This whole workflow is summarized in Fig. 2.

For the annotation of the literature, all the ontologies listed above were employed. Out of 24,559 abstracts, 21,035 of them were annotated, i.e., at least one term was found in their content (for terms appearing more than once in an abstract only one occurrence was considered). From the identified terms, those with low or rare frequencies were removed (<5 total hits across all searches). After preprocessing, this resulted in 2,399 unique terms from which terms related to nutrition were selected and considered for statistical analysis. It should be noted here that in the absence of any specific ontology available for nutrition related terms, with this study being the first of its kind, we sought to manually annotate all the available ontological terms using clinical and expert opinion, thus resulting in a list of 445 nutrition related terms given in the Table S3. Therefore, in the remainder of this paper, whenever we use the word “terms,” it is implicitly assumed that they are relevant to nutrition only.

### Statistical analysis

Statistical analysis was performed in R software. To account for the variation of the number of publications over time, the counts of each term found in a search for a pair of years for a specific disease condition, were adjusted with respect to the number of the papers published in literature for this condition and annotated from the workflow for this specific year (document-based normalization). To explore the significance of the variability of ontological terms between the disease conditions, the Vegan package (Oksanen et al., 2017) was used, particularly, the function *adonis* for PERMANOVA (ANOVA for distance matrices). Clustering between the disease groups, how dissimilar the terms for a given search (e.g., year or condition) are from each other and temporal changes in literature were assessed using the reduced-order representation of the datasets using the non-metric multidimensional scaling (NMDS), which reduces the multivariate dataset to two or more dimensions (similar to Principal Component Analysis (PCA)) based on dissimilarity (Bray–Curtis distance) between the terms for a given search. The Local Contributions to Beta Diversity (LCBD) was also used with a Hellinger transformation (Legendre & De Cáceres, 2013), where the overall beta diversity is divided into individual contributions from samples to identify outliers. The smaller the LCBD value is, the closer the sample is to the group average. To identify ontology-based terms that were significantly different between the conditions, *Kruskal–Wallis* test (Kruskal & Wallis, 1952) was used. The *Benjamini–Hochberg* correction was used on the returned *P*-values to correct for multiple testing and *Dunn’s* test as a post hoc procedure for pair-wise comparisons, where appropriate.

## Results

### Ontological terms clustered IBD separately from non-IBD conditions with temporal changes observed in the literature of each disease group

**Figure 3:**
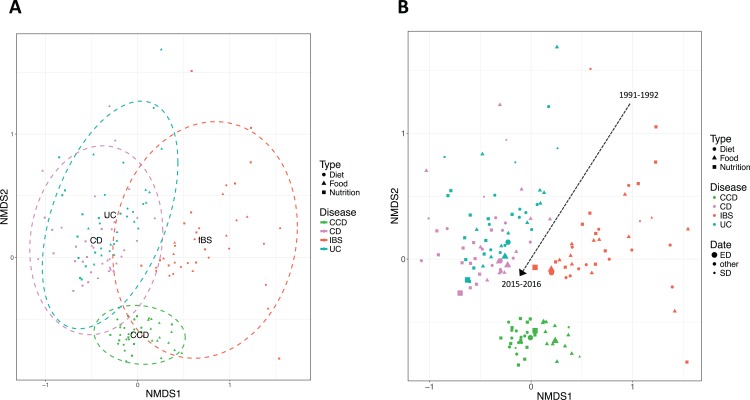
Non-metric multidimensional scaling (NMDS) based on Bray–Curtis distance demonstrating clustering of IBD and non-IBD groups in the 26-year timeline. Points indicate searches in pairs of years. (A) Ellipses describe 95% CI of standard deviation for a given group. (B) Dashed arrow represents transitions in the timeline. The size of the points corresponds to the date they describe where smaller size indicates earlier years and larger one more recent dates. Study sites: SD, starting date (1991–1992); ED, ending date (2015–2016); other, intermediate dates.

When the composition of the ontological terms for the disease conditions was assessed using NMDS plots, findings demonstrated an evident clustering of IBD related ontological terms distinct from non-IBD (Fig. 3A). The clusters for CCD and IBS stood well apart from those of CD and UC. CD and UC showed a degree of overlap, suggesting a degree of similarity in the ontological terms between these two conditions. Temporal variability was also noticeable from the NMDS plot (Fig. 3B). The beta diversity analysis revealed that the nutrition-related literature for each disease group has shifted over time. For all groups, the between-year variability was higher in the earlier dates, but gradually decreased, as we moved forward in time. This was clearer in the case of CD, UC, and IBS. It could be seen that the proximity between CD and UC was increasing more for the later years and that the two IBD groups were further converging to a similar set of ontological terms.

**Figure 4:**
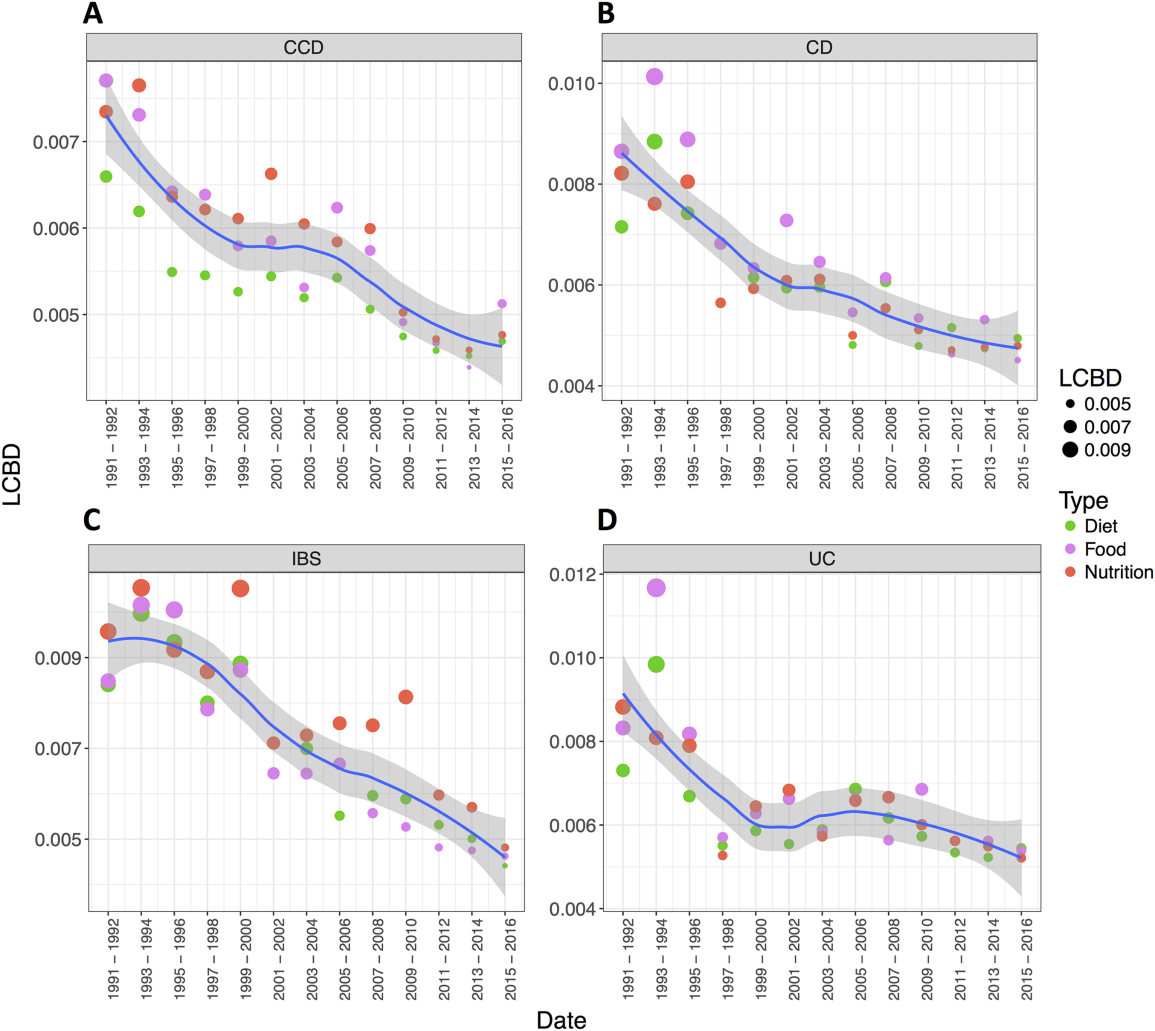
The relative contributions to beta diversity (LCBD) per disease condition. LCBD analysis demonstrating temporal variations in the literature of (A) CCD, (B) CD, (C) IBS and (D) UC (distances from group average). Loess curve with shaded 95% CI illustrates *t*. LCBD analysis demonstrating temporal variations in the literature of each disease group (distances from group average). Loess curve with shaded 95% CI illustrates the trends for each disease condition.

The convergence between the groups was also obvious when LCBD (Legendre & De Cáceres, 2013) analysis was applied. The findings, in this case, showed a decreasing trend of the LCBD values over the years for each disease group, more noticeable for the case of CD, UC and, IBS (Figs. 4A–4D). This indicated that the relative contribution of each sample (search for a pair of years) in every group was shifting towards the mean value (multivariate centroid) of the sample space when approaching more recent dates, suggesting their gradual convergence in recent years. This pattern indicated a relative consensus on a particular nutrition research theme for these disease conditions.

### Most frequent topics and conserved patterns in the literature of the disease conditions

**Figure 5:**
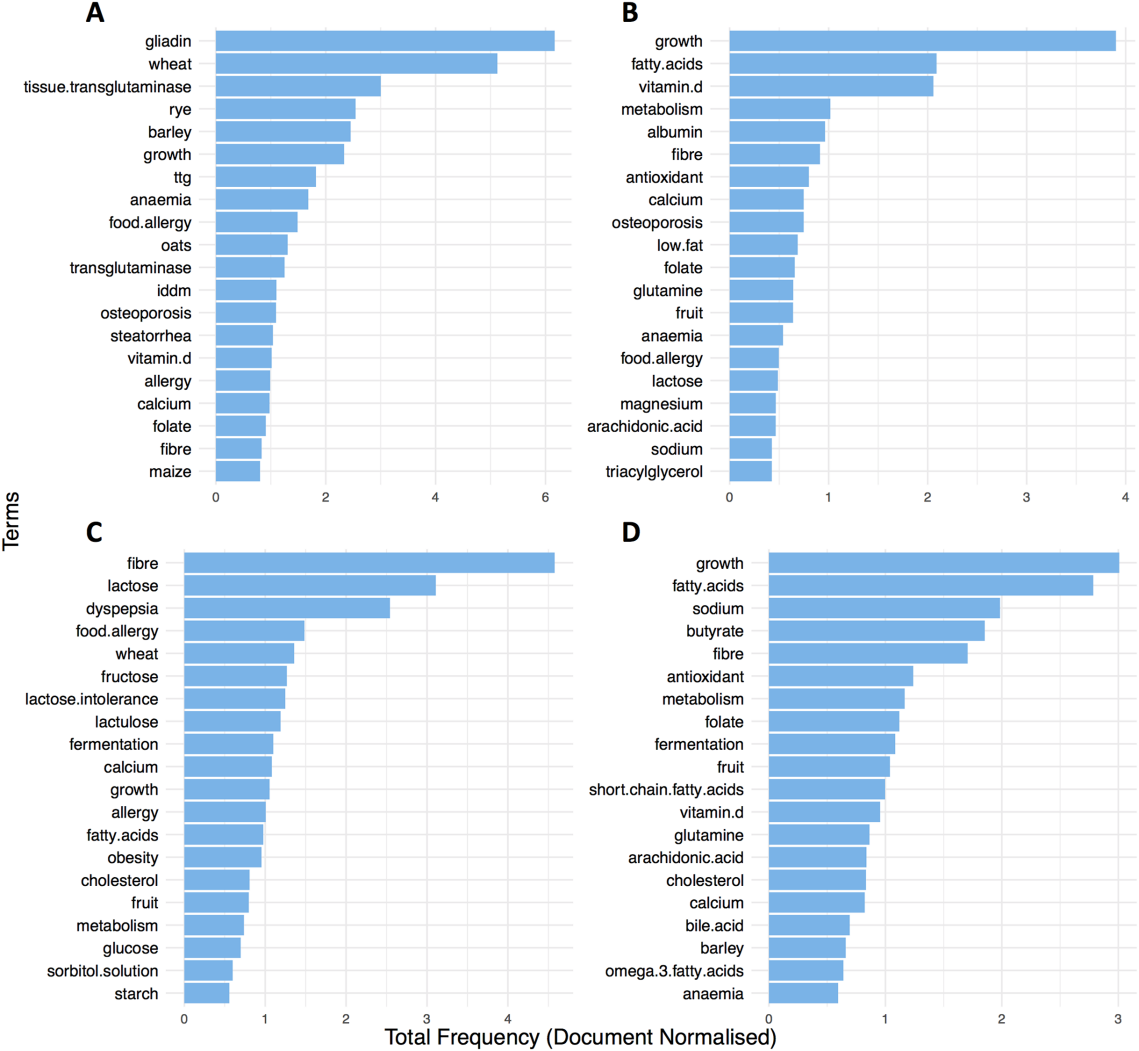
Top 20 most frequent ontological terms in the literature of (A) CCD, (B) CD, (C) IBS and (D) UC for the entire time frame (1991–2016).

Permutational multivariate analysis of variance (distances between groups) suggested that most of the variability was explained significantly by the different disease conditions (*R*^2^ = 27%, *p* = 0.001). To further explore this and inspect for terms that stratify the groups, we first looked at the twenty most frequent terms in the literature of each condition for the entire time frame. Findings showed that CD and UC, shared more than a half (65%) of their most common topics and terms such as *growth* (Freq. CD = 3.90; UC = 3.00) and *fatty acids* (Freq. CD = 2.08, UC = 2.78) were listed as the top two most frequent in the literature of the IBD groups (Figs. 5B and 5D). In a similar way, *wheat* (Freq. = 6.16) and *gliadin* (Freq. = 5.12) were unsurprisingly some of the most prevalent in the literature of CCD research (Fig. 5A). Likewise, the ontological terms *fibre* (Freq. = 4.57) and *lactose* (Freq. = 3.10) were found very common in IBS (Fig. 5C).

**Figure 6:**
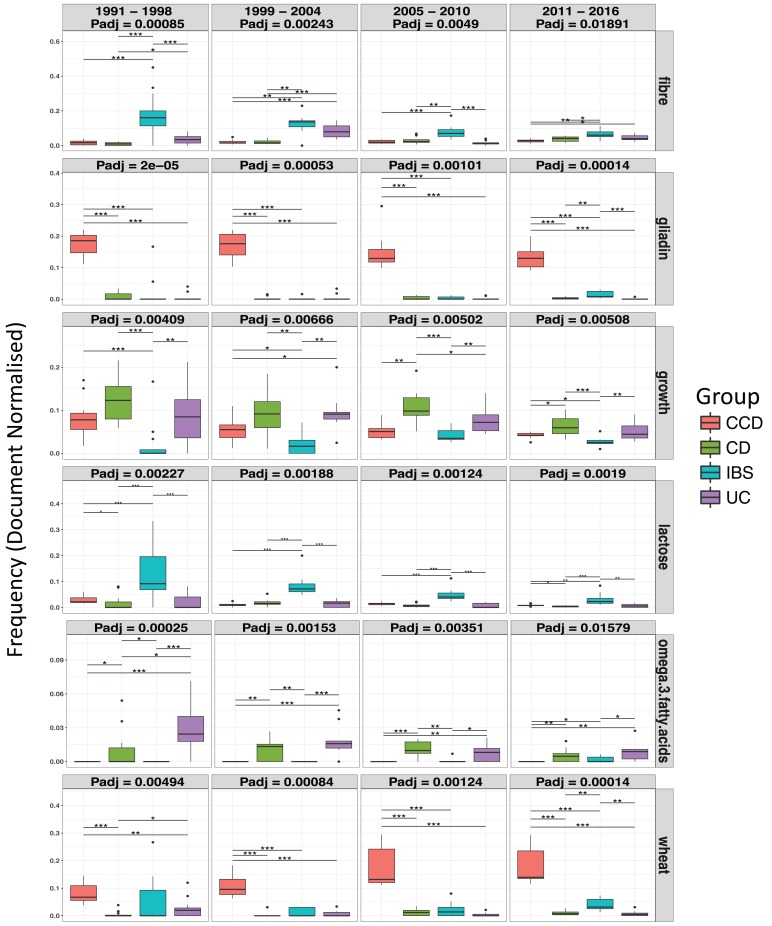
Ontological terms whose frequency differentiated between the disease groups, over separate subsets of time intervals. Box plots indicate the median, lower, and upper quartiles of the document-based normalized frequency obtained for a specific term from the searches performed over the dates of a time interval, across the nutritional categories: Nutrition, Food, and Diet, for a single group. Filled circles represent outliers. Dunn’s comparison with asterisks indicating significant differences * = *p* < 0.05, ** = *p* < 0.01, and *** = *p* < 0.001.

Moreover, differential analysis performed over separate time intervals (see Table 1) showed that the above findings were fairly conserved between the groups over the years (Fig. 6). This can suggest a continuous scientific interest for these topics in the research of each disease. In addition, results showed multiple terms becoming significant between the disease conditions for each time interval (*P*adj < 0.05; see Table 1 and Tables S1A–S1D). Specifically, in CCD, terms for *gliadin, wheat, rye, barley,* and *oats* were found to be stably frequent between 1991 and 2016 and clearly more common compared to the other groups (CCD > other diseases; Fig. 6 and Tables S1A–S1D). In a similar way, a considerable presence of *fibre* and *lactose* was observed in IBS throughout the years with findings also indicating a decrease in the frequency of both terms for the more recent dates (Fig. 6).

**Table 1:** Significance analyses performed on the identified ontological terms.

Number of ontological terms that differentiated over time in each disease condition			
Disease group	*n* = 445	Significant terms (*P*adj < 0.05)	Percentage (%)
	Subset size		
CCD	372	99	26.61
CD	385	185	48.05
IBS	287	169	58.88
UC	369	162	43.90

Number of ontological terms that differentiated between the disease conditions over separate time intervals			
Time interval	*n* = 445	Significant terms (*P*adj < 0.05)	Percentage (%)
	Subset size		
1991–1998	290	51	17.58
1999–2004	337	73	21.66
2005–2010	383	62	16.18
2011–2016	425	143	33.64

**Notes:**

Temporally changing terms were explored for each disease group individually (Subset size). Ontological terms becoming significant between the groups were also explored using differential analysis in separate time intervals. An adjusted *P*-value (*P*adj) < 0.05 was considered significant in each test. Percentage indicates the number of terms found significant over the size of the subset used for significance testing. *n* = total number of nutrition-related terms in the initial composite frequency table.

In the case of the IBD groups, terms such as *omega-3 fatty acids* and *n-6 fatty acids* were evidently more frequent compared to IBS and CCD where they were less common (CD and UC > CCD and IBS; Fig. 6; Tables S1B–S1D). For *omega-3 fatty acids*, the pattern was relatively stable over time (between 1991 and 2016) with some slight decrease for both CD and UC between 2011 and 2016 (Fig. 6). In a similar way, *n-6 fatty acids* were very common in CD and UC between 1999 and 2016 (Tables S1B–S1D). *Growth* term was also observed to be significantly different between the disease groups (Fig. 6). In CD, the same term had the highest prevalence with UC and CCD following respectively, appearing the least in IBS (CD > UC > CCD > IBS; Fig. 6). However, only during 1991–1998, this term appeared in CCD almost in similar levels to CD and UC literature.

### Ontological terms showing temporal changes in the literature of the disease groups

Analysis of variance using the *adonis* function showed that also temporal variability (expressed as in pairs of years) explained up to 19% of the changes in the use of ontological terms (*R*^2^ = 19.0%, *p* = 0.001). To investigate this further, differential analysis was performed on each term (see Table 1). Findings showed a number of terms differentiating over time in the literature of the disease conditions (*P*adj < 0.05; see Table 1 and Tables S2A–S2D).

**Figure 7:**
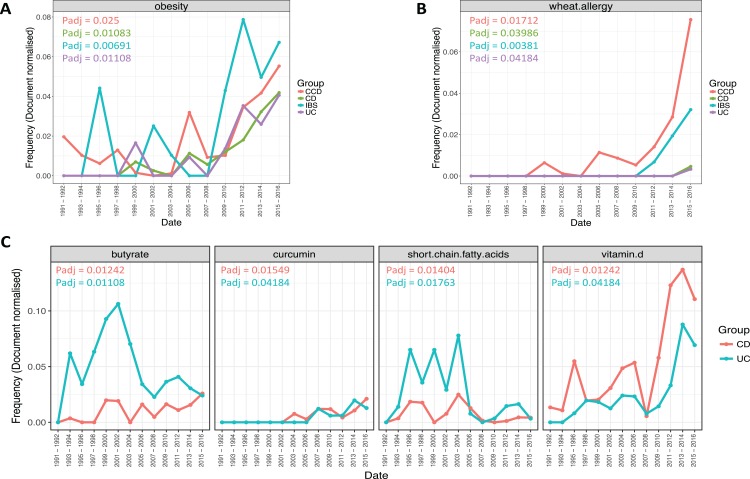
Trends of ontological terms whose frequency differentiated temporally in the literature of the gastrointestinal conditions. Plots (A) and (B) describe the prevalence over time of *obesity* and *wheat allergy* respectively, in all disease groups, and plot (C) describes the prevalence of terms found to differentiate over time in relation with CD and UC. Points indicate the mean document-based normalized frequency obtained for a specific term from a search conducted for a pair of years across the nutritional categories: Nutrition, Food, and Diet, for a single disease group.

More specifically, results revealed a considerable increase in the frequency of *obesity* term for all disease conditions (*P*adj CCD = 0.025, CD = 0.01083, IBS = 0.00691, UC = 0.01108; Fig. 7A). This was more evident after years 2008–2009, for each disease group. *Obesity* was higher in IBS between 2009–2010 and 2015–2016 compared to the other groups, with CCD being next and CD and UC following respectively. Similarly, *wheat allergy* was found becoming more common between the disease conditions over the years (*P*adj CCD = 0.01712, CD = 0.03986, IBS = 0.00381, UC = 0.04184; Fig. 7B). This term was noticed more frequent for CCD and IBS in the more recent dates (2011–2012 and thereafter). CCD seemed to be the group where *wheat allergy* was increasing the most with IBS being next. In the case of CD and UC, the same term was found to be equally prevalent between 2015 and 2016 for both groups, but clearly in a lower frequency when compared to the non-IBD types.

The frequency of several terms was also found to change temporally in relation to CD and UC (Fig. 7C). This was the case for *butyrate* (*P*adj CCD = 0.025, UC = 0.01108) and *curcumin* (*P*adj CCD = 0.01549, UC = 0.04184). *Butyrate* showed an increasing trend in the literature, most prominently in UC, with a peak frequency noticed in 2001–2002 and becoming considerably less common onwards (Fig. 7C). The same term was notably less common in CD compared to UC, where it became frequent between 1999–2000 and 2001–2002 and it was found in similar levels to UC in 2015–2016 (Fig. 7C). In addition, a partially transient prevalence over time was seen for the term *short-chain fatty acids* (*SCFAs*). *SCFAs* (*P*adj CCD = 0.01404, UC = 0.01763) were noticed to be more frequent for both groups between 1993–1994 and 2003–2004 and decreasing rapidly onwards, particularly in the case of UC (Fig. 7C). Moreover, the term *vitamin D* (*P*adj CCD = 0.01242, UC = 0.04184) was found more common in CD compared to UC and becoming frequent over the years for both groups showing a notable increase between 2009–2010 and 2013–2014 (Fig. 7C). However, after these dates, a slight decrease could be observed in both cases for the years 2015–2016.

## Discussion

In this study, we collated and assessed nutrition-related ontological terms from the literature of IBD and two other GI conditions. We inspected how certain nutrition terms differentiated between the groups and evolved in the scientific literature over the last 26 years. Results showed discriminating differences between IBD and non-IBD types and secular patterns in the literature of each disease separately. It was demonstrated that the terms related to the IBD types clustered distinctly from those of the non-IBDs. It was shown that the literature of each group was shifting over time and that it was gradually converging for the recent dates in the timeline. This was more evident for the case of CD and UC, but also noticeable for the other groups as well. This suggests that research topics are similar in the recent years for these diseases.

The prevalence of several terms that stratify the disease conditions in a conserved manner over time was also illustrated. More specifically, it was clearly noticed that terms describing gluten-related proteins and containing food, such as *gliadin*, *wheat*, *rye,* and *barley* were found in high frequencies in the literature of CCD. This was an expected outcome for CCD (McGough & Cummings, 2005) and suggests that our workflow is specific. Similarly, *fibre* was found to be considerably prevalent for IBS compared to the other groups. This observation aligns with studies suggesting that alteration of certain dietary *fibre* intake can be beneficial for this condition (El-Salhy et al., 2012) and a low FODMAP diet is now recognized as a successful management strategy for functional bowel disorders like IBS (Staudacher et al., 2011; Halmos et al., 2014). In the case of the IBD, terms such as *omega-3 fatty acids* and *n-6 fatty acids* were very common compared to IBS and CCD where their frequency was very low. This finding aligns to studies exploring the role of *omega-3* and *n-6 fatty acids* in the regulation of inflammation and as treatment modalities in IBD (Cabré, Mañosa & Gassull, 2012; Patterson et al., 2012; Barbalho et al., 2016), although their clinical efficacy is now less clear. In addition, the frequency of *growth* term appeared more prominently in the IBD groups compared to the other conditions and more evidently in the case of CD, where height deficits are more often compared with UC or IBS where delayed *growth* and short stature are less common (Gerasimidis, McGrogan & Edwards, 2011; Sigall-Boneh et al., 2017; Mason et al., 2017).

Patterns from temporal analysis revealed that *obesity* was steadily increasing in all groups and becoming very common in literature. This finding is in agreement with recent evidence from studies showing a growing prevalence of *obesity* in IBD patients (Flores et al., 2015) and mechanistic studies trying to unravel the role of adipose tissue in the inflammatory response (Wozniak et al., 2008; Bertin, Desreumaux & Dubuquoy, 2010). In the past, while malnutrition and inadequate nutrition in CD and UC patients were studied as the most common extra-intestinal complications in IBD, research seems to shift to studies looking at *overnutrition* and *obesity*.

On the contrary, a transient focus was demonstrated for *SCFAs* and particularly *butyrate*, in both UD and CD. *SCFAs* are well known and characterized bacterial metabolites produced from the fermentation of undigested fibre in the colon. The level of *SCFAs* content in fecal samples has been shown to be related to the pathogenesis of some GI conditions, including IBD (Venter, Vorster & Cummings, 1990). Among *SCFAs*, *butyrate* is the most extensively studied and several clinical studies document beneficial effects of *butyrate* but also issues with its production and colonic utilization in IBD (Scheppach et al., 1992; Steinhart et al., 1996). However, the frequencies of both these terms were found to become considerably lower, especially in the case of UC, for the more recent years reflecting a loss of interest in these topics in IBD research. This trend may represent the evolution of microbiome research in IBD from the role certain metabolites to the broader role of the microbiome and its broad metabolites, particularly now that OMICS technologies and computational power are more accessible. An interesting trend was seen for *vitamin D*. Despite the steady increase been observed for this term over time, a decrease of published interest has been noticed recently, in both IBD groups. This observation is likely to indicate an increase in the role of *vitamin D* in IBD pathogenesis, considering particularly the high prevalence in this population, which has recently declined in the absence of consistent evidence implicating this vitamin as an environmental risk factor for autoimmune diseases like CD (Narula & Marshall, 2012). The decrease found in the recent years hence may suggest that less clinical attention is now given to the role of *vitamin D* in IBD or that this certain research theme has been exhaustively studied.

## Conclusions

We have presented a rapid, automated workflow for the systematic annotation of scientific literature with rich metadata employing a broad range of domain ontologies. We have applied this tool for the identification and analyses of ontological terms in certain GI diseases and nutrition-related literature. Although automated interpretation cannot completely replace human judgement, it can save significant time to process very large amounts of literature, free from reviewer’s bias and can reduce this information to a far more comprehensive and manageable set of deducable patterns from which it is easier to draw conclusions. Application of summary statistics, regularly used in environmental microbiology, allow description of differences between multiple conditions and patterns over time within a certain condition. The current workflow is applicable to any type of literature and can perform equally for any kind of published data accessed from PubMed database. However, the manually developed nutrition-only ontology library used in this study highlights the need to develop theme specific ontology libraries that can make the workflow more effective and more efficient.

## Supplemental Information

10.7717/peerj.5047/supp-1 Supplemental Information 1 Table S1. Differential expression analysis of nutrition-related terms between disease conditions. Four tables, Table_S1A (1991–1998), Table_S1B (1999–2004), Table_S1C (2005–2010), and Table_S1D (2011–2016) for differential expression analysis of nutrition-related terms between diseases using Kruskal-Wallis test. Only those terms are shown where the adjusted *P*-value (*P*adj) < 0.05. Mean expression indicates the mean document-based normalised frequency obtained for a specific term for each disease group. A post hoc pairwise Dunn’s comparison indicating significant differences between the groups is shown on the right half.

10.7717/peerj.5047/supp-2 Supplemental Information 2 Table S2. Differential expression analysis of nutrition-related terms between years. Four tables, Table_S2A (CCD), Table_S2B (CD), Table_S2C (IBS), and Table_S2D (UC) for differential expression analysis of nutrition-related terms between years using Kruskal-Wallis test. Only those terms are shown where the adjusted *P*-value (*P*adj) < 0.05. Mean expression indicates the mean document-based normalised frequency obtained for a specific term for each interval.

10.7717/peerj.5047/supp-3 Supplemental Information 3 Table S3. Subset of ontological terms related to nutrition which were manually selected using clinical and expert opinion and were considered for statistical analysis in our study.

## Glossary

**Table:**

Terminology	Description	Usefulness	References
Alpha diversity	Reflects the within-sample diversity.	Inspect how many different individuals e.g., microbial species could be detected in one sample.	–
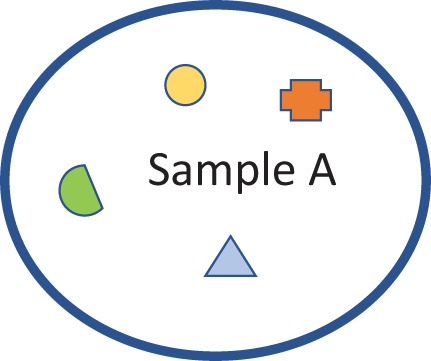
Beta diversity	Reflects the between-sample diversity.	Inspect dissimilarities (distance and/or clustering) between samples.	–
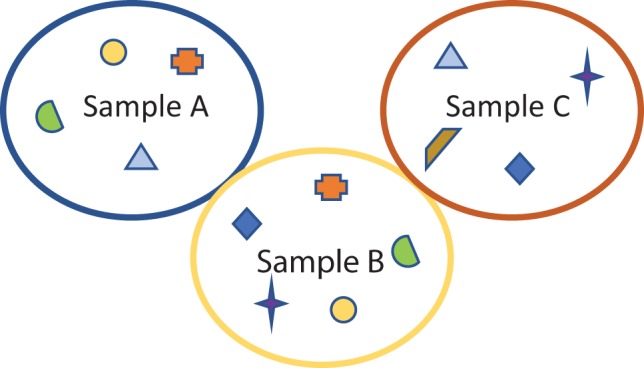
Kruskal–Wallis test	Test whether the medians of two or more groups are equal.	Determine if there are statistically significant differences between multiple groups (two or more).	R’s *stats*: kruskal.test()
Local contributions to beta diversity (LCBD)	The overall beta diversity is divided into individual contributions from samples. Smaller the LCBD value, the more closer the sample is to the group average.	Inspect how far or close are the individual contributions from samples to the group average.	–
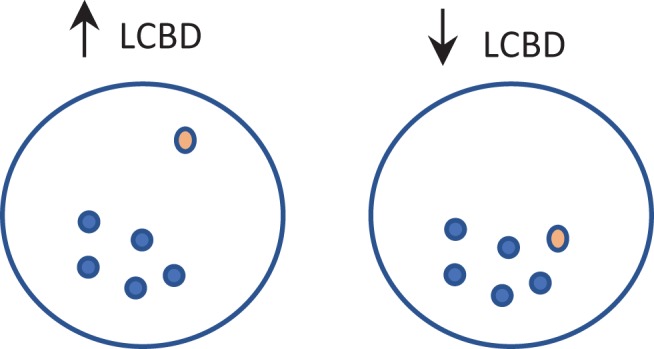
Non-metric multidimensional scaling (NMDS)	Ordination technique where data from multiple dimensions (e.g., from multiple communities, sites, etc.) are simplified into just a few and represented as points in a 2D space (similar to PCA).	Inspect beta diversity of a multivariate dataset in a 2D space.	R’s *vegan*: metaMDS()
	N: sites, S: species		
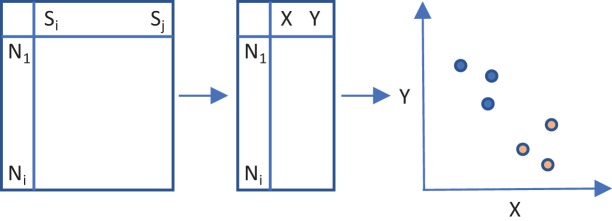
Ontology	A formal specifications of a list of terms that are arranged in a hierarchical structure with a unique ID assigned to a term including its’ synonyms. A term itself can be a part of multiple hierarchies and *n*-ary relations pattern with these terms often collectively available as an OBO file.	Create a consensual vocabulary of terms.	https://owlcollab.github.io/oboformat/doc/GO.format.obo-1_4.html
Permutational multivariate analysis of variance (PERMANOVA)	Compare groups of objects and test if there are differences in the position and/or spread, in a multivariate space, of the compared groups attributes.	Measure effect size and significance on beta diversity for a grouping variable.	R’s *vegan*: adonis()

## Additional Information and Declarations

### Data Availability

The following information was supplied regarding data availability: The code for pyTag workflow and the associated data are available at: https://github.com/KociOrges/pytag.
